# Factors affecting belongingness of Occupational Therapists in an Interprofessional Clinical Environment

**DOI:** 10.12669/pjms.42.5.13539

**Published:** 2026-05

**Authors:** Nazish A Qadir, Ahsan Sethi

**Affiliations:** 1 Nazish A Qadir Institute of Physical Medicine and Rehabilitation, Khyber Medical University, Peshawar, Pakistan; 2 Ahsan Sethi Health Professions Education, Department of Public Health, College of Health Sciences, QU Health, Qatar University, Doha, Qatar

**Keywords:** Belongingness, Interprofessional learning, Occupational Therapists

## Abstract

**Objective:**

Belongingness ensures psychological safety, tacit knowledge sharing, and interprofessional collaboration. Unfortunately, the role of Occupational Therapists (OTs) is often misunderstood, which affects their professional identity, job satisfaction, and sense of belongingness. This study explores the factors affecting the belongingness of occupational therapists in an interprofessional clinical environment.

**Methodology:**

A qualitative case study was conducted from January 2022 to 2023. A purposive maximum variation sample of 10 OTs across Pakistan was invited to participate. An interview guide was developed using an ‘Integrative Belonging Framework’, and online semi-structured interviews were conducted. Thematic analysis was performed using inductive and deductive coding.

**Results:**

Professional competence, interprofessional collaboration, motivation, professional identity crises, and organizational barriers were identified as factors affecting OTs’ sense of belongingness in an Interprofessional clinical environment.

**Conclusion:**

Occupational Therapists’ professional competence, motivation, and interprofessional collaborations contribute to their belongingness. Professional identity crises and organizational barriers negatively impact their belongingness. A competency-based curriculum, provision of resources, interprofessional learning, and collaborative practices can foster a strong sense of belongingness.

## INTRODUCTION

Humans need to feel a sense of belongingness to innovate, excel in their future goals, and find meaning in life. Belongingness refers to the feeling of being connected, valued, or accepted by others during collaboration with individuals and surroundings.^1^ According to Maslow’s hierarchy of needs, a sense of belongingness becomes essential after satisfying the basic needs, such as food, shelter, and sleep. The feelings of self-worth and belonging demand a secure, understanding, and inclusive environment, and the same needs to be reflected in workplaces. Belonging to a workplace is vital for learning, a safe environment, tacit knowledge sharing, and inclusive practices. In contrast, the lack of belongingness potentially impacts learning, motivation, and well-being services, and engagement in the workplace.^2^

Occupational Therapists (OTs) are an important member of the healthcare team, who facilitate health and rehabilitation in patients or clients with disability, injury, or illness. The role ranges from physical to psychological, hospital to community-based, habilitation to rehabilitation, and neonatal to geriatric care.^3^ They work in a variety of healthcare settings including schools, nursing centers, rehabilitation centers, mental health facilities, daycares, and hospitals. OT services are beneficial for the promotion of health and well-being among various chronic illnesses, cancer survivors, postpartum depression secondary prevention of diseases, pain management, and long-term hospitalization. However, the role of OTs is misconceived by other health professionals, and in turn, their services are not effectively **utilize**d as needed.^4^ These misconceptions and a lack of appreciation affect their job satisfaction, professional identity, engagement and sense of belongingness as OT in an interprofessional clinical environment.^5^,^6^

In the literature, many studies explored the factors influencing the sense of belongingness among undergraduate medical, nursing, physical therapy and occupational therapy students.^7^-^10^ There has been no research on the factors that impact OTs sense of belongingness as members of an interprofessional healthcare team in the clinical environment. This study explores the factors affecting the belongingness of occupational therapists in an interprofessional clinical environment.

## METHODOLOGY

This qualitative case study was conducted from January 2022 to 2023. A case here was defined as the interprofessional clinical environment in which Occupational Therapists practice. A case study approach was considered most appropriate for examining contextual, organizational, and relational influences on belongingness among occupational therapists working in interprofessional practice. An ethical approval was granted by the Institute of Health Professions Education & Research (1-9/IHPER/MHPE/KMU/23-05; dated February 20, 2023).

The participants included Occupational Therapists (OT’s) working in the tertiary care hospitals of Pakistan. A purposive maximum variation sample of 10 OT’s was iteratively selected and interviewed until saturation was achieved. Maximum variation sampling was achieved by selecting participants from different cities across Pakistan, from both public and private sectors, and from diverse clinical settings including pediatric, adult, and psychiatric units.

For belongingness, we considered an Integrative Belonging framework, which defines belongingness as a feeling and experience that arises from four interlinked constituents: competencies, opportunities, motivation, and perceptions supported by the social system in which a person lives. They interact and have an impact on one another, shift, progress, and adapt as the individual passes over different experiences and contexts of social, temporal, and environmental systems.^11^

The Interview guideline was developed using the Integrative Belonging framework, which explored OTs’ motivation, perceptions, and opportunities to connect with in an interprofessional team. The interview guide was validated by experts (n=8) and then piloted (n=2) to ensure comprehensiveness. The interviews were semi-structured and conducted in English.

Data analysis followed Braun & Clarke’s six steps of thematic analysis.^12^ initially; the transcripts were read thoroughly for initial codes. Deductive coding was done by using an integrative framework of belongingness, whereas the inductive coding allowed new insights from the data. The inductive insights refined the preliminary deductive coding structure. All the codes were sorted into categories to develop subthemes. The researchers continuously reviewed codes and subthemes to ensure that they accurately reflect data. Finally, the themes were identified from the subthemes through continuous discussions.

Credibility was ensured through prolonged engagement and rapport building with the participants. A clear methodological and analytical description along with the use of the Integrative Belonging Framework helped ensure dependability and confirmability. A description of occupational therapists and their working context has been provided to ensure transferability. Member checking was not performed as qualitative data analysis is an interpretive process in which the researcher may bring different perspectives to the analytic process.^13^

## RESULTS

Most of the participants were females. The OTs worked across different provinces of Pakistan and had more than three years of clinical experience (Table-I). Our analysis revealed eleven subthemes grouped into five key themes: professional competence, motivations, interprofessional collaborations, professional identity crises and organizational barriers.

**Table-I:** Participants Characteristics.

Characteristics	Frequency	Percentage
***Gender***		
Male	02	20%
Female	08	80%
***Age***		
29-35	06	60%
40-44	04	40%
***Qualification***		
Bachelors	07	70%
Masters	03	30%
***Experience***		
<5	01	10%
5-10	04	40%
>10 years	05	50%
***Workplace***		
Sindh	06	60%
Punjab	03	30%
Khyber Pakhtunkhwa	01	10%

### Professional Competence:

**1A:*Communication skills:*** OTs emphasized that effective communication skills are essential in building a strong team and enable them to feel a sense of belongingness within the team. *“For a strong team and feeling belonged, good communication skills are crucial” (P1)* A few OTs mentioned that due to their inadequate communication skills, they are unable to discuss their ideas. *“I have the Knowledge, but sometimes I feel a little hesitant to talk about things due to my limited communication skills.” (P2)*
***1B:Attaining Clinical Experience:*** Occupational Therapists shared that gaining clinical experience helped them develop skills and feel more connected to the team. *“The advantage of working in a team is that you learn from people with time and experience and start to feel belong to the team (P1)”* Another OT shared that: *With the passage of time and experience, I believe I now feel more belonged. I have developed skills and confidence to work.” (P10)*

### 2) Motivations:

***2A: Intrapersonal factors:*** Few Occupational Therapists were of the view that regardless of the environmental factors, it is one’s inner drive that enables them to belong to their surroundings. *“Belongingness is pretty much personal; It mostly depends on the individual’s perception of their surroundings.” (P1)* Few Occupational Therapists believed that their drive to help others is an important factor in fostering their sense of belonging in an interprofessional team. *“I belong to my team because we all have one goal which is to help people.” (P4)*
***2B:Sense of Achievement:*** Several OTs expressed that they feel a sense of belonging when they observe improvements in their patients. *“Seeing progress in the patients is what makes me feel an important part of the team, i believe it is amazing to be a part of someone’s healing journey.” (P9)* Majority of the participants shared that observing positive results in patients create a sense of shared achievement and goal among team members: *“Improved patient prognosis has always helped me to connect to the people more in the team. (P6)”*
***2C:Acknowledgment from the Team members:*** Most occupational therapists emphasize that feeling appreciated by their colleagues and patients is an influential factor in cultivating their belonging within a team. *“ We are present at every departmental meeting. Our colleagues always appreciate our work, which makes us feel a valued part of the team.” (P3)* One of the Occupational therapists shared their experience of feeling an improved sense of belonging upon receiving appraisal from colleagues. *“It was last year we had a seminar, and I presented on the role of occupational therapy in pediatric mental health seminar. I received appreciation from my department … This experience provided me with an improved sense of belongingness. (P1)*

### 3: Inter-Professional Collaborations:

***3A:Inter-Professional Meetings:*** Most participants expressed that regular formal meetings have a positive impact on their learning and strengthen professional bonds with the team members. *“We have a meeting twice a week with our team members where each member discusses their role in the presented cases. These meetings enhance our learning and strengthen our professional* *relationship.” (P7)* Few participants mentioned difficulty connecting due to the lack of regular formal meetings. “*We cannot connect or discuss with each other as we don’t have time for any discussions in our schedule. Usually, we are working in silos”. (P7)*
***3B:Informal Discussions:*** In addition to the formal discussions within the department, a few OTs stressed upon informal meet-ups and get-togethers. According to them, such informal conversations allowed them to cultivate strong connections with the team members. *“Our bonding is not just limited to formal meetings. We have some other get-togethers as well. These gatherings provide opportunities to come together in a more relaxed setting, and we feel like a family” (P1)* Other participants feel a stronger connection when they meet outside the formal meetings: *“We connect more if we sit and have a good time with team members other than the case discussions and professional meetings.”(P6)*

### 4: Professional Identity Crises:

***4A:Low awareness levels:*** Occupational Therapists have expressed concern over insufficient knowledge of team members regarding the role of Occupational Therapy in various domains, which negatively impacts their sense of belonging. *”Some of the professionals don’t refer patients. They do not consider OT because of a lack of knowledge and this somehow integrates an inferiority complex.” (P10)* One participant highlighted the discrepancies in understanding the OT roles among health professionals in different domains. *“The therapists working in pediatric or psychiatry setups feel more belonged compared to working in other domains. This is because the professionals in pediatric settings have a better understanding of the role of Occupational therapists.”*. (P7)
***4B:Overlapping Roles of Team Members:*** Most of the OTs stressed that while working in an interdisciplinary team the roles of an occupational therapist often overlap with the other members of the team, affecting their harmony with the team. *“Most of the time our roles overlap with physical and speech therapists. This creates challenges for team togetherness and belonging” (P3)* Some OTs perceived that other professionals in the team did not let them work in their domain. *“They believe we are only limited to fine motor activities and that they can perform tasks within our domain” (P10)*.

### 5: Organizational Barriers:

***5A: Unavailability of administrative seats:*** From the viewpoint of some Occupational Therapists, the unavailability of administrative seats for OT has a potentially negative impact on the sense of belonging to the team. *“How can we feel belong? We do not have the same opportunities and administrative positions as others. (P5)*
***5B:Limited Resources:*** OTs highlighted the inadequate allocation of resources for OT services. “*I believe they have access to more resources and privileges due to their primary authority, which we lack. We are not equally valued. “(P7)* Some of the occupational therapists were of the view that OTs are not paid well for their jobs and are provided with fewer resources, giving them the feeling of not being an equal part of the team. “*One of the main reasons that demotivates occupational therapists is the insufficient salary and fewer resources. It leads to a sense of underperformance and not fitting in”*.

## DISCUSSION

This study explored the factors influencing Occupational Therapists’ sense of belongingness in an interprofessional clinical environment. The findings focus on how professional competence, motivation, and interprofessional collaboration contribute to belongingness rather than just describing individual or organizational attributes. Professional identity crises and organizational barriers negatively impact their belongingness. These elements interact with the less resourced and hierarchical system, relevant to the middle and low-income countries.^14^In addition to this, it is aligned with how limited resources, lack of standardized competency frameworks, and time constraints are barriers for the allied health professionals in the physician-led leadership hierarchical system of Pakistan.^15^

Professional competence emerged as one of the core contributors to belongingness. Participants expressed how attaining skills and experiential learning boosted their confidence and acceptance among colleagues. There are similar findings in literature specific to the context of high-income countries that skill learning contributes to the confidence of allied health professionals.^16^ On the contrary in LMICs, where healthcare professionals face barriers to their continues education and professional development in terms of resources, lack of acceptance among other stake holders.^17^ Correspondingly, this aligns with the evidence suggesting that knowledge sharing and cooperation in the workplace serve as a mediator of belongingness in the context of rehabilitation settings and have positive effects on patients’ outcomes and the broader healthcare system.^18^

Good communication skills are necessary for gaining professional competence while working in a team of professionals with diverse backgrounds. Lack of evidence-based knowledge, inability to understand English, and the use of discipline-specific terminologies also become barriers to effective communication among team members. Studies reported that occupational therapists experienced challenges when they had difficulties in communication, whereas good communication with patients brought a sense of satisfaction.^14^

Motivation appeared to be an essential element for belongingness in a clinical environment. OTs highlighted Intrapersonal factors and sense of achievement as important factors in achieving motivation. Likewise in health care institutions of Pakistan, intrinsic motivations are essential for ensuring the psychological needs of an individual and have positive outcomes on both the individual and organizational levels.^19^ The participants also attributed appreciation from colleagues and patients as a motivating factor. Support and welcoming gestures of others in the clinical working environment help foster belongingness.^20^

Literature has identified continuous formal and informal encounters as significant factors towards learning and team collaboration at all levels. While most occupational therapists acknowledged the constructive influence of team discussions on their belongingness, participants also stressed the increasing workload and time consumed during formalized interactions. Hence, there is a need to ensure a balance between clinical workload and formal meetings or informal meetings.^21^

Professional identity crises and organizational barriers negatively impact their belongingness. A strong professional identity aids OTs in upholding professional standards and adapting to demanding work environments.^22^ However, the lack of knowledge of the OTs role among other healthcare team members is a barrier to their belongingness. There is a lack of knowledge about OT domains and practices among other professionals and the general public, which has an impact on their professional identity.^23^ Another concern is the overlapping roles of OT with other rehabilitation team professionals. Organizational barriers, including the unavailability of administrative positions and a lack of resources, appeared to be a pervasive factor affecting participants’ belongingness in our study. The lack of resources prompts feelings of being challenged, frustration, and inadequacy among OTs. Moreover, the discrimination among various professionals in a healthcare team at the workplace has an impact on motivation and self-efficacy. The accreditation bodies should emphasise on the provision of adequate resources and leadership roles for OTs.^24^

Our findings suggest that fostering a sense of belongingness has a significant impact on professional identity, well-being, and workplace performance. Hence, there is a need to incorporate interprofessional learning in undergraduate and postgraduate education of OTs. There is also a need to emphasize role identification and communication skills, along with clinical skills. Implementing a robust mentoring program with counselling sessions during undergraduate training may strengthen OT students’ professional identity and help equip them with the resilience needed for the workplace.^25^ The employers should ensure work-life balance, a safe learning environment in the workplace with inter-professional collaborations, clearly defining the roles of each team member, and promoting awareness. Involving OTs in management positions may also help remove barriers to their sense of belongingness.

It is a qualitative, multi-center study providing real-life experiences of Occupational Therapists. This study addresses the regional gap in the LMIC setting. OTs across Pakistan have shared their individual experiences, offering practical implications for education, practice, and policy.

### Limitations:

There was limited representation from two of the provinces in Pakistan. Moreover, the interviews were taken online, and the lack of face-to-face interaction may have impacted the rapport-building and in-depth exploration of their experiences. However, the richness of our findings suggests otherwise. Future research should involve a focus group among interprofessional teams, including OTs, for further insight into the professional identity of OTs.

## CONCLUSION

Occupational Therapists’ Professional competence, motivation, and interprofessional collaborations contribute to their belongingness. Professional identity crises and organizational barriers negatively impact their belongingness. A competency-based curriculum, provision of resources, interprofessional learning, and collaborative practices can foster a strong sense of belongingness.

### Recommendations:

Future studies should include the perspectives of other team members, and representations of professionals from other regions, as well as longitudinal studies to observe the development of belongingness over time.

## Availability of data and materials:

The datasets analyzed during this study are available from the first author upon reasonable request.

### Authors’ Contribution:

**AS and NAQ** conceived and designed the study. **NAQ** collected the data. Both authors were involved in data analysis and manuscript writing.Both authors performed the final review and approved the manuscript.
